# Veratrum in Pneumonia

**Published:** 1888-03

**Authors:** J. T. Hutchins

**Affiliations:** Oakland, Texas


### CORRESPONDENCE.

### VERATRUM IN PNEUMONIA OF CHILDREN.

*Editors Daniel Texas Medical Journal:—*

Enclosed please find two dollars, which you will be kind enough to place to my credit. The more I read Daniel's Texas Medical Journal, the more do I become impressed with its intrinsic value. I was greatly interested in the article from the pen of Dr. L. M. Stroud, of Forney, that appeared in the November number of 1887,on the value of veratrum in the treatment of pneumonia. I had occasion to test its worth in a case of pneumonia recently in a little girl, age eleven years, with an acute attack of the right lung. She had been suffering three days before I was called to see her. I saw her first in the morning; she was then very restless with a hot dry skin, cheeks flushed, pulse full but frequent, temperature high, respiration hurried and accompanied with the usual characteristic nervous cough. After obtaining the objective and subjective symtoms in the case, I directed a blister to the side over seat of pain and gave a dose of calomel. I then began at once with the veratrum, not with a seven-drop dose, as the doctor suggested, but five-sixth of a drop, giving it myself the first day every half-hour or hour, as I thought best, in a teaspoonful of cold water, until I had administered seven drops of the tincture of veratrum. The admirable effects of the drug in this case were plainly seen in calming the patient, in exciting the skin to a gentle perspiration and lowering the temperature. It brought the pulse down to the normal volume. It diminished the respiration and in union with this lessened the nervous cough. Suffice it to say, the veratrum was continued from my first day's visit up to the eleventh day of illness in five-sixth of a drop dose every hour, whenever the febrile movement was heightened or highly flushed cheeks were noted.

On the twelfth day the stage of resolution set in with a steady improvement, thence on with my patient. In this case I used no quinine, but gave veratrum preference with the view of testing its merits.

I remain, Very Respectfully,

J. T. Hutchins.

Oakland, Texas.

